# Poisonous Wall Paper

**Published:** 1874-04

**Authors:** 


### POISONOUS WALL PAPER.

TAKE a small piece of light green wall paper, let a drop of nitric acid fall on it, then apply some spirits of hartshorn; if the color changes to deep blue, copper is present. When the fumes have nearly disappeared, apply a solution of nitrate of silver, three grains to the ounce; if arsenic is present, a yellow ring is formed which becomes more distinct when dried. This easily applicable test is furnished by Mr. Wm. L. Billin, of Philadelphia.

